# “THERE IS A SHORTAGE AND MISUSE OF TERTIARY NURSING”: PERSPECTIVES OF NURSING ASSISTANTS IN ACUTE CARE

**DOI:** 10.1093/geroni/igad104.3157

**Published:** 2023-12-21

**Authors:** Jennifer Wagner, Vivian Miller, Lauren Maziarz, Melissa Burek, Julia Bell, Kaley Perry

**Affiliations:** Bowling Green State University, Bowling Green, Ohio, United States; Bowling Green State University, Bowling Green, Ohio, United States; Bowling Green State University, Bowling Green, Ohio, United States; Bowling Green State University, Bowling Green, Ohio, United States; Bowling Green State University, Bowling Green, Ohio, United States; Bowling Green State University, Bowling Green, Ohio, United States

## Abstract

The primary role of nursing assistants (CNAs) in the acute care setting is to provide direct care to patients. As frontline workers, they are often the first to report changes in condition to the licensed staff. The U.S. Bureau of Labor Statistics projects an increased employment need for nurse aides over the next ten years. However, the annual rate of nurse aide turnover is over 120 percent. A study conducted in 2015 by Brady suggests that there is a 36% turnover rate nationwide in acute care. Guided by a socio-ecological model, qualitative findings from 17 nurse aides (N=17) reveal interpersonal factors, such as relationships with others at the workplace and relationships with patients, as impacting turnover. Institutional and Community factors were also found to impact turnover, including staffing levels, resources, education, and compensation. Informed by the interview findings, a quantitative study was created. CNAs (N=230) from a variety of health care settings participated. Twenty-five percent were from the acute care system (N=56). More than seventy percent (N= 40) have considered leaving their employment. The top two reasons were pay (N=15) and to pursue other things (N=9). The top two work challenges were providing direct care to bariatric patients (N=28) and not enough supplies (N=13). The favorite part of working in acute care is getting to know the patients (N=26). Little research has been conducted on the turnover of CNAs in the acute care setting. CNAs are vital to caring for patients in the acute care system.

